# Distinct Bile Acid Profiles in Patients With Chronic Hepatitis B Virus Infection Reveal Metabolic Interplay Between Host, Virus and Gut Microbiome

**DOI:** 10.3389/fmed.2021.708495

**Published:** 2021-10-04

**Authors:** Zeyu Sun, Chenjie Huang, Yixian Shi, Rusha Wang, Jun Fan, Ye Yu, Zhehua Zhang, Kundan Zhu, Minwei Li, Qin Ni, Zhi Chen, Min Zheng, Zhenggang Yang

**Affiliations:** ^1^State Key Laboratory for Diagnosis and Treatment of Infectious Diseases, National Clinical Research Center for Infectious Diseases, Collaborative Innovation Center for Diagnosis and Treatment of Infectious Disease, School of Medicine, The First Affiliated Hospital, Zhejiang University, Hangzhou, China; ^2^Kidney Disease Center, Shulan (Hangzhou) Hospital, Hangzhou, China; ^3^Department of Hepatology, Mengchao Hepatobiliary Hospital of Fujian Medical University, Fuzhou, China

**Keywords:** chronic hepatitis B, metabolomics, bile acid metabolism, gut microbiome, NTCP

## Abstract

Hepatitis B virus (HBV) can hijack the host bile acids (BAs) metabolic pathway during infection in cell and animal models. Additionally, microbiome was known to play critical role in the enterohepatic cycle of BAs. However, the impact of HBV infection and associated gut microbiota on the BA metabolism in chronic hepatitis B (CHB) patients is unknown. This study aimed to unveil the distinct BA profiles in chronic HBV infection (CHB) patients with no or mild hepatic injury, and to explore the relationship between HBV, microbiome and BA metabolism with clinical implications.

**Methods:** Serum BA profiles were compared between CHB patients with normal ALT (CHB-NALT, *n* = 92), with abnormal ALT (CHB-AALT, *n* = 34) and healthy controls (HCs, *n* = 28) using UPLC-MS measurement. Hepatic gene expression in CHB patients were explored using previously published transcriptomic data. Fecal microbiome was compared between 30 CHB-NALT and 30 HCs using 16S rRNA sequencing, and key microbial function was predicted by PICRUSt analysis.

**Results:** Significant higher percentage of conjugated BAs and primary BAs was found in CHB patients even without apparent liver injury. Combinatory BA features can discriminate CHB patients and HCs with high accuracy (AUC = 0.838). Up-regulation of BA importer Na+ taurocholate co-transporting peptide (NTCP) and down-regulation of bile salt export pump (BSEP) was found in CHB-NALT patients. The microbial diversity and abundance of *Lactobacillus, Clostridium, Bifidobacterium* were lower in CHB-NALT patients compared to healthy controls. Suppressed microbial bile salt hydrolases (BSH), 7-alpha-hydroxysteroid dehydrogenase (hdhA) and 3-dehydro-bile acid Delta 4, 6-reductase (BaiN) activity were found in CHB-NALT patients.

**Conclusion:** This study provides new insight into the BA metabolism influenced both by HBV infection and associated gut microbiome modulations, and may lead to novel strategy for clinical management for chronic HBV infection.

## Introduction

Human hepatitis B virus (HBV) infection remains a major public health problem, affecting over 250 million population globally (1). Unfortunately, there was no curable option for HBV infection. Series of recent discoveries have revealed complex interplay between HBV infection and host bile acids (BA) metabolism which may lead to future antiviral therapies.

The Na+ taurocholate co-transporting polypeptide (NTCP, encoded by SLC10A1), originally known as a major hepatic transporter for BAs (2–4), was recently discovered as the receptor permitting the hepatotropic entry of HBV (5–7). This interesting dual role of NTCP played in both HBV entry and BA transportation was further confirmed by a report showing inhibition of taurocholate uptake by HBsAg pre-S1 polypeptide in NTCP-expressing HepG2 cells (8). Furthermore, HBV was found able to induce the key rate-limiting enzyme for BA synthesis, cholesterol 7α-hydroxylase (CYP7A1), possibly via a FXRα-dependent fashion (9–11). Normally, the hepatic BA metabolism is tightly regulated by a group of factors orchestrated by FXRa which was kept activated by BA binding. The active FXRa is required for the expression of conjugation enzymes UGT2B4 and SULT2A1 (12, 13), detoxification enzymes ADH1A/B (14) and CYP3A4 (15), efflux pumps BSEP and ABCB4 (16), while conversely, suppresses CYP7A1, NTCP and FXRa itself via a SHP-mediated negative feedback loop (17, 18). In HBV infection cell models, inadequate hepatocellular BA level due to NTCP obstruction by HBV binding can lead to inactivation of FXRa which in turn up-regulates CYP7A1, NTCP and FXRa (11). Moreover, the inactive FXRa appeared to be a proviral factor that can transcriptionally enhance the HBV activity (10, 11). Therefore, the competitions between HBV and BAs for host factors occur both in viral entry and post-entry stages, and HBV is capable of hijacking BA regulatory network for its own benefit. Additionally, the host inflammatory response to the HBV activity also modulates the BA metabolism, as excessive cytokine activities was found to suppress NTCP and leads to cholestasis and jaundice in CHB patients (19–21). Therefore, caution should be taken to investigate the interaction of BA metabolism and HBV in patients with exacerbated hepatic inflammation.

Other than the interplay between HBV infection and BA metabolism in the liver, one cannot ignore the increasingly recognized role of gut microbiota (22). The enterohepatic circulation of BAs within the gut-liver axis requires microbial biotransformation before BA being reabsorbed into the portal vein (22–24). This process depends on intestinal bacteria containing bile salt hydrolases (BSHs) which transformed conjugated BA (CBAs) to unconjugated forms (UCBAs), as well as dehydrogenase and oxidoreductases which convert primary BAs into their secondary counterparts (23). Importantly, the links between liver diseases and gut microbiota have been widely documented (25–27). Particularly, fecal samples from CHB patients were found to have a marked decrease in bifidobacteria and lactic acid bacteria (25). However, little was known whether such modulation of gut microbiota could influence the BA metabolism in CHB patients.

The potential clinical implications of BA modulation in HBV infected patients are multifold. Bile acids play essential roles in glucose and cholesterol homeostasis, intestinal absorption of nutrients (28). In addition, BAs activate a variety of nuclear receptors and signaling pathways (29). Abnormal accumulation of BAs could cause hepatic and biliary injury and inflammation, which are associated with fibrosis and cirrhosis eventually leading to liver failure or liver cancer (30–34). Therefore, BA metabolism has been advocated as a novel therapeutic target for liver diseases treatment (35–37). However, comprehensive profiles of BAs in liver disease at very early or mild stages of CHB are still lacking.

This study aimed to investigate the association between HBV infection, microbiome modulation and BA metabolism in CHB patients with mild hepatic inflammation. We first compared the fasting serum profiles of BA in healthy controls and CHB patients with or without hepatic inflammation. For the first time, we show distinct pattern of BAs can be found in CHB patients even with normal ALT level. On top of this, the roles of hepatic genes and gut bacteria related to BA metabolism were investigated in CHB patients.

## Materials and Methods

### Patients Enrollment

A total of 126 HBV-infected patients and 28 healthy subjects were recruited from the First Affiliated Hospital, School of Medicine, Zhejiang University (Hangzhou, China). Routine biochemical parameters including aspartate transaminase (AST), alanine transaminase (ALT), alkaline phosphatase (ALP), γ-glutamyltransferase (GGT), creatinine (Cr), albumin (ALB), γ-globulins (GLB), total bilirubin (T-Bil), direct bilirubin (D-Bil), prothrombin time (PT), blood urea nitrogen (BUN), uric acid (UA), triglyceride (TG), total cholesterol (TC), low-density lipoprotein (LDL), glucose (GLU), serum HBV-DNA and hepatitis viral antigens (HBsAg, HBeAg) were measured in the clinical laboratory of the First Affiliated Hospital, School of Medicine, Zhejiang University. Chronic HBV infection is defined as HBsAg sero-positive status for at least 6 months according to 2015 APASL guidelines (38). The upper limit of normal laboratory reference (ULN) of ALT was 40 IU/mL (38). Exclusion criteria including: (1) cases complicated with other infectious diseases or liver diseases such as alcoholic hepatitis, non-alcoholic fatty liver disease, biliary diseases, liver cancer, liver cirrhosis, liver failure; (2) patients with history of using UDCA supplements, or drugs lead to cholestasis such as ademetionine and silymarin, antibiotics or probiotics or herbal medicine within 24 weeks; (3) cases with gastrointestinal tract abnormalities; (4) pregnant or lactating women. Detailed definitions for clinical criteria can be found in Supplementary Table 1. This study was carried out with the approval of the Ethics Committee of the First Affiliated Hospital, School of Medicine, Zhejiang University, and all subjects have signed informed consent. Since liver injury due to inflammation was known to alter BA profiles, we therefore divide CHB patients into two subgroups, CHB-NALT with normal ALT level (<40 IU/mL), and CHB-AALT with abnormal ALT level (≥40 IU/mL) indicating hepatic injury.

### Serum Bile Acids Measurement

Serum samples were obtained using their fasting blood in the early morning. To extract circulatory BAs, 50 μL of serum samples were allowed to thaw on ice and were subsequently spiked with 150 μL cold acetonitrile. Samples were vortexed 3 × 10 s and maintained at −20°C for 20 min to precipitate proteins. After centrifugation at 10,000 g for 10 min at 4°C, 100 μL supernatants were transferred to clean tubes and dried in a Speed Vac concentrator (Labconco). The residue was reconstituted in 50 μL of 50% methanol: water and was centrifuged at 10,000 g for 10 min at 4°C.

The following 15 most common human BA species were determined by a LC-MS/MS method: cholic acid (CA), chenodeoxycholic acid (CDCA), deoxycholic acid (DCA), lithocholic acid (LCA), ursodeoxycholic acid (UDCA), glycocholic acid (GCA), glycochenodeoxycholic acid (GCDCA), glycodeoxycholic acid (GDCA), glycolithocholic acid (GLCA), glycoursodeoxycholic acid (GUDCA), taurocholicacid (TCA), taurochenodeoxycholic acid (TCDCA), taurodeoxycholic acid (TDCA), taurolithocholic acids (TLCA), and tauroursodeoxycholic acid (TUDCA). BAs were separated by Waters ACQUITY UPLC BEH C18 column (2.1 mm × 10 cm, 1.7 μm, 130 Å) installed in a 1,290 UHPLC system (Agilent) at a flow rate of 0.5 mL/min. The BA profile was analyzed by an Xevo TQ-S mass spectrometer (Waters) operated under the multiple reaction monitoring (MRM) mode as detailed in the Supplementary Materials. Data acquisition parameters for each BA species in MRM experiment can be found in Supplementary Table 2. The typical chromatogram can be found in (Supplementary Figure 1).

### BA Related Gene Expression Analyses

Expression level of 31 hepatic genes related to BA metabolism (Supplementary Table 3) from CHB patients and healthy controls (dataset GSE83148) (39) were downloaded from NCBI GEO database. The affymetrix chip-based expression signals were log10 transformed prior to statistical analysis.

### Fecal Microbiome Profiling

Fecal samples of 30 CHB-NALT patients and 30 healthy volunteers (Supplementary Table 4) were used for 16S rDNA profiling. Detail about sample preparation, 16S rDNA library building and sequencing, OTUs assembly, and microbial community diversity analyses can be found in Supplementary Materials. To predict bile salt hydrolase gene content in the samples, sequencing data were mapped to OTUs defined in the 13_5 release of the Greengenes database. Relative abundances of those OTUs were used to predict the abundances of genes corresponding to KEGG orthology K01442 (cholylglycine hydrolase), K00076 (7-alpha-hydroxysteroid dehydrogenase, hdhA), K07007 (3-dehydro-bile acid Delta4, 6-reductase, baiN) with PICRUSt algorithm (40).

### Statistical Analyses

Variables following a normal distribution were presented as mean ± SEM and were compared by parametric *t*-test. BA profiles and ratios did not fit normal distribution according to the Shapiro-Wilk test (all *p* < 0.05), therefore were compared by nonparametric Mann–Whitney U test. Logistic regression models were built by stepwise method, using *P* < 0.05 for entering the model and *P* > 0.10 for removing from the model. Area under the curve (AUC) in the receiver operating characteristic (ROC) analysis was used to estimate the predictive power of indicators. All statistical tests were two-sided, and *P* < 0.05 were considered as statistically significant. The statistical analyses were performed by using R (v3.2.0).

## Results

### Clinical and Laboratory Characteristics of the Patients and HCs

Characteristics of the study subjects are summarized in Table 1. Compared to healthy controls, CHB-NALT patients had significantly higher levels of serum GLB, ALT, AST, and TBIL levels (all *p* < 0.05). The other biochemical parameters of CHB-NALT patients and healthy controls were within the reference ranges. In comparison, CHB-AALT patients had significantly higher level of ALT, AST, GGT, and TBIL (all *p* < 0.05) than CHB-NALT patients as expected. There was no difference of TP, ALB, ALP, Cr, BUN, UA, TG, TC, LDL, and GLU levels among all groups.

**Table 1:** Baseline characteristics of study subjects for serum examination.

	**HC (*n* = 28)**	**CHB-NALT (*n* = 92)**	**CHB-AALT (*n* = 34)**
Gender (male/female)	14/14	52/40	24/10
Age (years)	46.00 ± 10.73	39.78 ± 10.56*	40.35 ± 11.61
HBV-DNA (copies/mL)	/	3.96 ± 10.99 E+07	1.70 ± 4.96 E + 07
HBsAg (IU/mL)	/	15408.15 ± 26749.88	5711.73 ± 13339.52
HBeAg (PEIU/mL)	/	89.38 ± 140.82	62.74 ± 119.83
TP (g/L)	72.62 ± 3.19	73.96 ± 4.69	72.74 ± 6.14
ALB (g/L)	48.13 ± 2.72	47.33 ± 2.99	46.00 ± 5.58
GLB (g/L)	24.51 ± 2.23	26.64 ± 3.72*	26.74 ± 3.84^#^
ALT (U/L)	15.46 ± 7.99	22.33 ± 8.35*	123.18 ± 143.59^#†^
AST (U/L)	18.36 ± 4.09	21.76 ± 6.38*	66.12 ± 54.04^#†^
ALP (IU/L)	69.54 ± 44.98	67.43 ± 18.63	75.94 ± 25.98^#^
TBIL (μmol/L)	11.11 ± 4.88	14.27 ± 7.95	23.85 ± 40.56^#^
GGT (U/L)	27.68 ± 23.12	19.08 ± 11.37	57.00 ± 37.82^#†^
Cr (μmol/L)	71.93 ± 13.04	72.30 ± 16.70	72.74 ± 14.57
BUN (mmol/L)	5.26 ± 1.35	4.87 ± 1.04	5.00 ± 0.98
UA (μmol/L)	294.54 ± 71.79	308.95 ± 80.74	311.44 ± 90.15
TG (mmol/L)	1.41 ± 1.35	1.18 ± 0.67	1.13 ± 0.53
TC (mg/dL)	4.71 ± 0.77	4.34 ± 0.93*	4.11 ± 1.07^#^
LDL (mg/dL)	2.58 ± 0.72	2.49 ± 0.68	2.27 ± 0.80
GLU (mmol/L)	5.05 ± 1.21	5.04 ± 0.70	5.33 ± 1.67

All data were presented by mean ± SEM. HC, healthy control; CHB-NALT, ALT normal patient; CHB-AALT, ALT abnormal patient; The p values of categorical and quantitative variables were determined by χ^2^ and Mann–Whitney U test. Significant difference (P < 0.05) between HC and CHB-NALT^*^, HC and CHB-AALT^#^ or between CHB-NALT and CHB-AALT were indicated.

### The Distinct Bile Acid Profiles in Chronic Hepatitis B Patients

**Figure 1:**
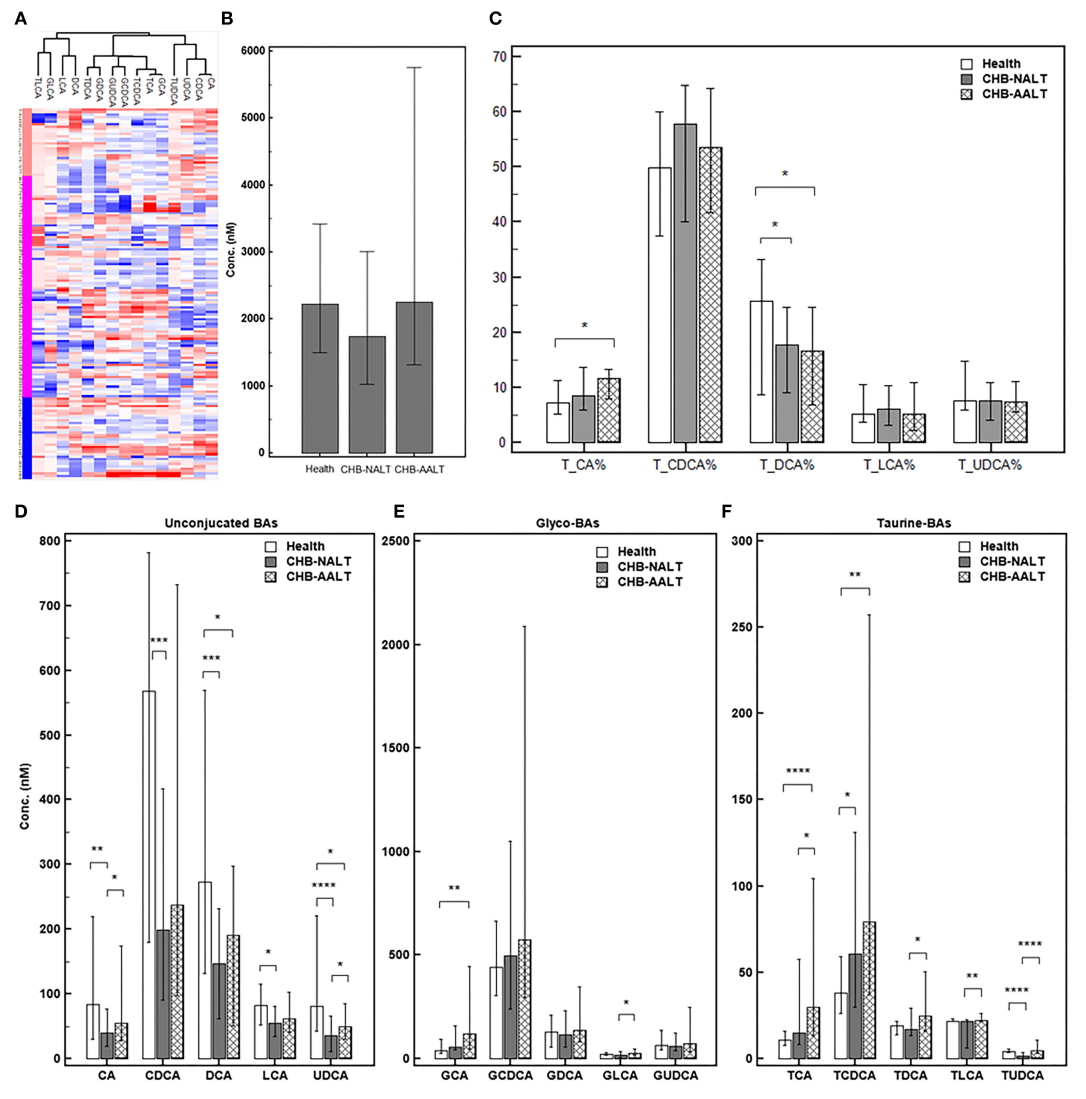
Summary of BA profiles. **(A)** Bile acid profiles across all samples were visualized by heatmap. Concentration of each BA in Log2 scale were transformed into Z-score by columns. The columns were clustered using the K-mean algorithm. The levels of **(B)** total BA, **(C)** percentage of total CA, CDCA, DCA, LCA and UDCA, and the levels of **(D)** each unconjugated, **(E)** glyco-conjugated and **(F)** taurine-conjugated BAs measured in different groups were summarized. Error bars represented 25–75% quartile. *P* values were determined by the Mann–Whitney *U* test, significant differences were noted by *p < 0.05, **p < 0.01, ***p < 0.001, ****p < 0.0001. CHB-NALT: ALT normal patient group, CHB-AALT: ALT abnormal patient group.

The overall BA profiles across all subjects are visualized in Figure 1 and summarized in Table 2. In brief, BA profiles were highly heterogeneous in CHB patients (Figure 1A). Although there was no significant difference in total BAs levels (Figure 1B, all *p* > 0.05), alteration of BA composition can be found among all three groups. Significantly, the percentage of total DCA, adding both unconjugated and conjugated forms, was lower in both CHB groups (Figure 1C). Lowest median level of all UCBAs was found in CHB-NALT patients, while highest median level of all UCBAs was found in healthy controls (Figure 1D). Gradual increases of conjugated forms of primary BAs, i.e. GCA, TCA, GCDCD, TCDCA were observed in CHB-NALT and CHB-AALT patients (Figures 1E,F). In addition, higher GLCA, TDCA, TLCA and TUCDA levels were found in CHB-AALT patients than CHB-NALT (Figures 1E,F).

**Table 2:** The BAs concentrations in serum measured in different groups.

**BA (nM)**	**HC**	**CHB-NALT**	**CHB-AALT**
CA	153.68 ± 159.64	78.06 ± 99.87*	197.71 ± 402.73^†^
CDCA	654.78 ± 660.43	329.35 ± 391.39*	518.80 ± 675.81
DCA	402.76 ± 350.11	163.01 ± 116.83*	219.69 ± 176.24^#^
LCA	94.95 ± 57.21	82.65 ± 107.05*	85.16 ± 70.34
UDCA	130.83 ± 114.96	67.53 ± 182.53*	76.23 ± 72.59^#†^
GCA	72.86 ± 93.69	145.75 ± 205.31	611.88 ± 1957.62^#^
GCDCA	649.19 ± 648.55	897.40 ± 1087.79	2405.02 ± 5935.38
GDCA	156.39 ± 149.88	163.27 ± 154.70	249.83 ± 298.31
GLCA	25.26 ± 15.82	32.06 ± 51.98	34.18 ± 23.41^†^
GUDCA	128.04 ± 178.12	148.76 ± 433.65	1379.11 ± 5822.72
TCA	18.56 ± 29.40	505.00 ± 2451.09	280.77 ± 1083.32^#†^
TCDCA	54.28 ± 69.18	226.64 ± 749.23*	704.94 ± 2707.97^#^
TDCA	24.62 ± 22.68	27.34 ± 31.35	42.62 ± 51.00^†^
TLCA	22.82 ± 19.29	24.06 ± 28.99	26.81 ± 13.82^†^
TUDCA	6.24 ± 5.63	14.62 ± 62.54*	268.95 ± 1427.16^†^
Total CBA	1140.60 ± 1057.12	2164.78 ± 3176.61	5967.35 ± 18853.30
Total UCBA	1436.99 ± 930.21	720.59 ± 635.21*	1097.60 ± 1073.55^#^
Total primary BA	1603.35 ± 1155.52	2182.19 ± 3068.23	4719.12 ± 11560.92
Total secondary BA	974.24 ± 503.56	703.18 ± 713.98*	2345.83 ± 7268.26
Total BA	2577.59 ± 1460.36	2885.37 ± 3312.46	7064.95 ± 18767.43

Table represent the mean±SEM. HC, healthy control group; CHB-NALT, ALT normal patient group; CHB-AALT, ALT abnormal patient group. P values were determined by Mann–Whitney U test, significant difference (P < 0.05) between HC and CHB-NALT*, HC and CHB-AALT^#^ or between CHB-NALT and CHB-AALT were indicated, respectively.

**Figure 2:**
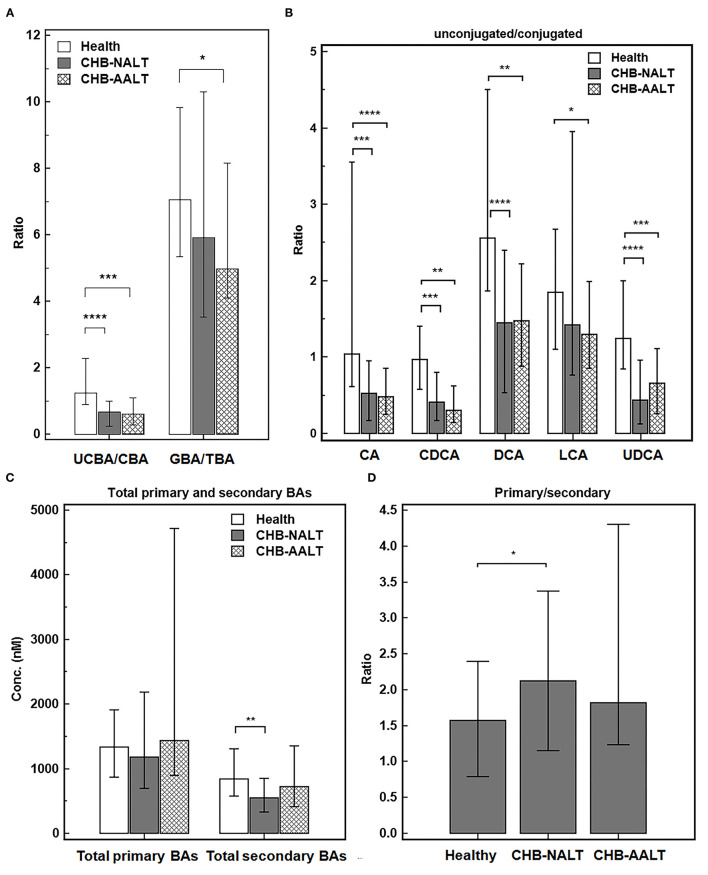
Alteration of BA ratios in CHB patients. The ratios of **(A)** total unconjugated BAs vs. total conjugated BAs, and total glyco-conjugated vs. total taurine-conjugated BAs, **(B)** ratios of unconjugated vs. conjugated individual BAs, **(C)** total primary BAs vs. secondary BAs levels and **(D)** ratios of total primary BAs vs. secondary BAs across all three groups were summarized. Error bars represented 25–75% quartile. *P* values were determined by the Mann–Whitney *U* test, significant differences were noted by *p < 0.05, **p < 0.01, ***p < 0.001, ****p < 0.0001. CHB-NALT: ALT normal patient group, CHB-AALT: ALT abnormal patient group.

Despite the heterogeneity of individual BA profiles across different groups, the relatively concerted change of unconjugated and conjugated BA groups prompted us to investigate the relevance of UCBA/CBA ratios with CHB progression. We found the total UCBA/CBA ratios were significantly lower in CHB-NALT and CHB-AALT patients than in HCs (both *P* < 0.001, Figure 2A). Moreover, the ratio of glycine/taurine-conjugated BAs gradually decreased from NCs to CHB-NALT and CHB- AALT patients. Similar trends can be found in UCBA/CBA ratio for each individual BA species (Figure 2B). For CHB-NALT patients, decreasing UCBA/CBA ratio compared to HCs can be found in CA, CDCA (both *P* < 0.001), DCA and UDCA (both *P* < 0.0001). For CHB-AALT patients compared to NCs, decreasing UCBA/CBA ratio can be found in CA (*P* < 0.0001), CDCA, DCA (both *P* < 0.01), LCA (*P* < 0.05) and UDCA (*P* < 0.001).

In addition to changes in UCBA/CBA ratios, we also identified modulation of primary/secondary ratios of BAs particularly in CHB-NALT patients. Although no significant changes of total primary BAs were found among three groups, total secondary BAs were significantly lower in CHB-NALT patients as compared to HCs (*P* < 0.01, Figure 2C), which correspondingly led to significantly higher total primary/secondary BA ratios in CHB-NALT patients (*P* < 0.05, Figure 2D). Moreover, compared to individual BAs that generally showed limited value to indicate CHB (Supplementary Table 5), we found total unconjugated BA (UCBAs) level, and its ratio to the conjugated BAs (UCBAs/CBAs), as well as total secondary BAs (SBAs) level have high AUC value (all >0.7) to distinguish CHB from healthy controls (Supplementary Figure 2A). Moreover, we found the ratio of unconjugated/conjugated CA, CDCA, DCA and UDCA also have higher AUC value (all >0.7) than individual BA species (Supplementary Figure 2B). These results then encouraged us to further build a combinatory model including total UCBAs, UCBAs/CBAs ratio and U/C ratio of CDCA by logistic regression (Supplementary Table 5) with higher diagnostic ability (AUC = 0.838, Supplementary Figure 2C) than conventional markers such as ALT, AST, ALP, GGT and TBIL (Supplementary Table 5). These results indicated BA based signatures can be used in junction with current biochemical markers to monitor CHB progress in very early stages.

### Modulated Expression of Hepatic Genes Related to BA Transport and Synthesis in CHB Patients

**Figure 3:**
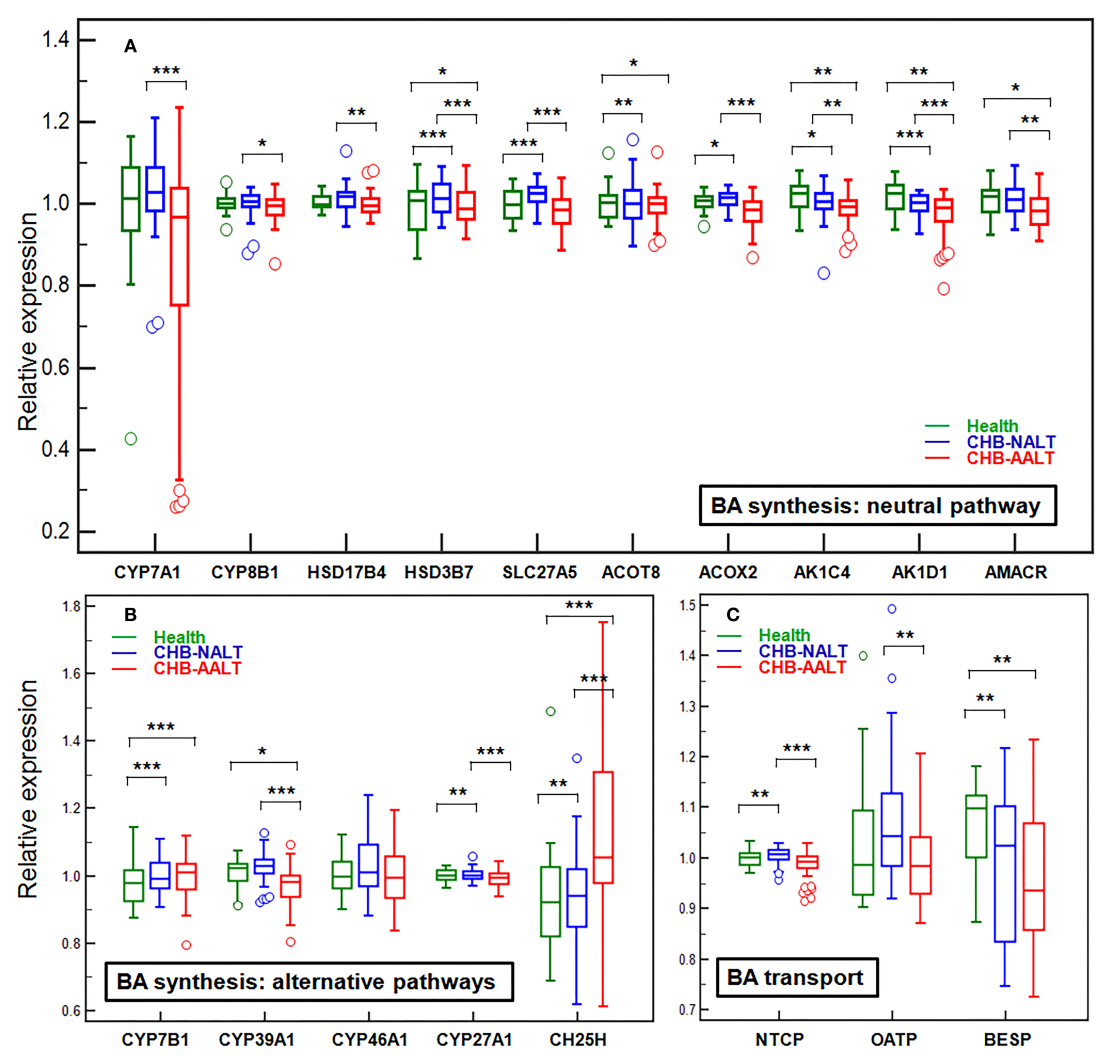
Alteration of hepatic genes related to BA transport and synthesis in CHB patients. The expression levels across all three groups were compared for genes related to *de novo* synthesis by **(A)** main neutral pathway, **(B)** alternative acidic or 24/25-hydrolyase pathways and **(C)** BA transport. All expression levels were normalized against the medium level. Error bars represented 25–75% quartile. *P* values were determined by the Mann–Whitney *U* test, significant differences were noted by *p < 0.05, **p < 0.01, ***p < 0.001. CHB-NALT: ALT normal patient group, CHB-AALT: ALT abnormal patient group.

Previously documented liver transcriptomic data comparing NALT-CHB and AALT-CHB patients with healthy controls (GEO dataset GSE83148) (39) were reanalyzed for genes related to BA transport and hepatic *de novo* synthesis (Figure 3). As expected, BA importer NTCP but not OATP was shown upregulated in CHB-NALT patients, but both were suppressed in AALT-CHB patients. Surprisingly, the key rate-limiting enzyme CYP7A1, only shown slight upregulation in CHB-NALT patients without statistical significance, but was found significantly inhibited in AALT-CHB patients (Figure 3A). On the contrary, its counterpart in the alternative pathways, CYP7B1, was found overexpressed in CHB patients (Figure 3B). Significant upregulation of none-rate-limiting genes, ACOT8, ACOX2, HSD3B7, SLC27A5, CH25H, CYP27A1 and CYP8B1 in the neutral pathway of BA synthesis were also recorded in CHB-NALT patients. Almost all synthesis related genes, except CH25H, were down-regulated in CHB-AALT patients. Enzymes related to BA conjugation, bile acid-CoA synthase (BACS) or bile acid-amino acid transferase (BAAT) did not show significant changes in CHB patients. Downregulation of BA exporter BESP was found in both NALT and AALT patients (Figure 3C).

### Distinct Gut Microbiome Profiles in Patients With CHB-NALT

**Figure 4:**
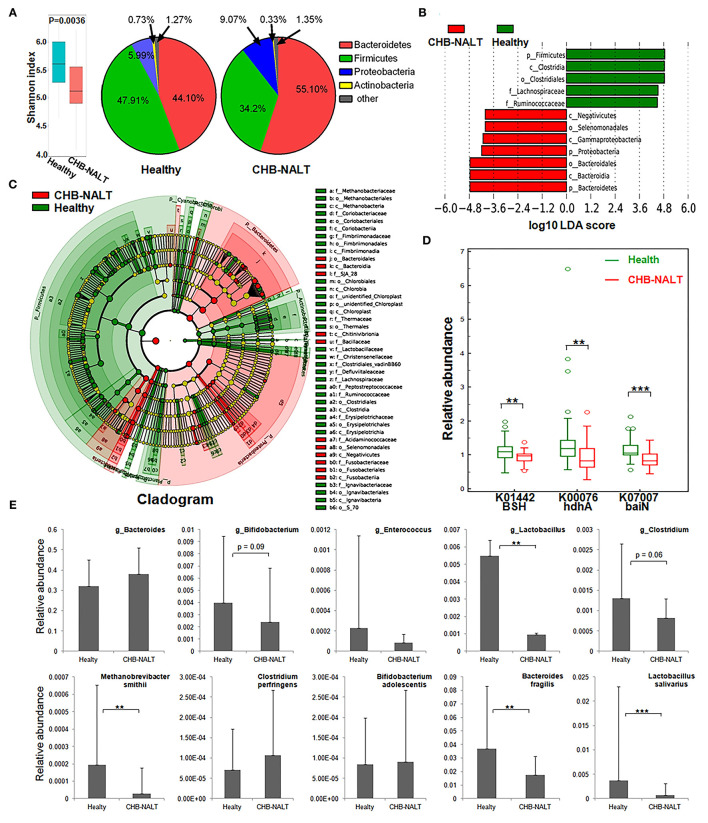
Modulation of fecal microbial profiles and functionalities related to CHB-NALT. **(A)** Shannon index of microbial ecological diversity and microbiota constituents at phylum level shown difference between groups. Linear discriminant analysis (LDA) coupled with effect size measurements identifies the taxons most differentially abundant between the groups. **(B)** Taxa with LDA significant cutoff >4 were shown. **(C)** Taxa with LDA > 2 were summarized by cladogram. **(D)** Taxa overrepresented in CHB-NALT (red) or healthy controls (green) were highlighted according to LEfSe analysis. The predicted gene abundance of BSH (KEGG orthology K01442), hdhA (K00076) and baiN (K07007) between the groups. **(E)** The relative abundances of BSH containing genus (top panel) and bacteria species (bottom panel) between the groups. *P* values were determined by the Mann–Whitney *U* test, significant differences were noted by *p < 0.05, **p < 0.01, ***p < 0.001. Data were presented by median ± quantiles.

To explore possible roles of microbiome related to the changes of BA composition in the early stage CHB, fecal microbiota profiles from CHB-NALT patients and healthy controls were analyzed by 16S rRNA sequencing. Overall, the analyses revealed significant decreased ecological diversity, as measured by Shannon index, in CHB-NALT (Figure 4A, *P* < 0.01). Analysis of bacterial population at the phylum level revealed that *Proteobacteria* and *Bacteroides* increased, while *Firmicutes* decreased in CHB-NALT patients compared to HCs (Figure 4A). Using LDA score > 4, the LEfSe analysis revealed that CHB patients had more *Bacteroidales* which belongs to *Bacteroidia, Selenomonadales* which belongs to *Negativicutes*, and *Gammaproteobacteria* which belongs to *Proteobacteria*, but had less *Lachnospiraceae* and *Ruminococcaceae* which both belong to class *Clostridia* and phylum *Firmicutes* (Figure 4B). Using LDA score > 2, the LEfSe analysis further provided more detailed list of 192 OTUs altered in CHB-NALT with their evolutionary tree summarized by cladogram (Figure 4C).

To reveal possible links to the distinct BA profiles in CHB-NALT patients, we first focused on BSH-harboring bacteria genera documented by previous studies, such as *Lactobacillus, Bifidobacterium, Enterococcus, Clostridium*, and *Bacteroides* spp (23, 41–44). Compared to HCs, the relative abundances of *Lactobacillus*, particularly the *Lactobacillus salivarius* was significantly lower in CHB-NALT (Figure 4E). Bifidobacterium and Clostridium were also shown decreasing level in CHB-NALT patients albeit with less statistical significance (*p* < 0.1). Despite that *Bacteroidia* was more abundant, one key BSH containing member *Bacteroides fragilis* (43) was found significantly suppressed in CHB-NALT patients (Figure 4E). In addition, the only member of archaeon harboring BSH activity, *Methanobrevibacter smithii* (44), was also found significantly lower in CHB-NALT patients. Predicted by PICRUSt analysis, BSH gene abundance in the CHB-NALT group was significantly lower than that of the HCs (Figure 4D, *P* = 0.006). These results indicated that the decreased microbial BA deconjugation activity might have contributed to the relatively higher percentage of UCBA in CHB-NALT patients. Analogously using PICRUSt estimation, we showed two critical secondary BA conversion enzyme groups: hdhA and baiN gene abundance was significantly lower in the CHB-NALT groups (Figure 4D, *P* = 0.006, *P* = 0.0006, respectively), which can be partly attributed to the decreasing level of class *Clostridia*.

## Discussion

Bile acids (BAs) play an important role in a wealth of physiological and pathological processes. Most prominently, bile acids are also key regulators of energy expenditure, glucose and lipid metabolism, thyroid hormone signaling, and cellular immunity (23, 45). The enterohepatic BA cycle is vital to the intestinal absorption of lipids and fat-soluble vitamins, and the elimination of cholesterol. Bactericidal BAs also play role to maintain healthy gut microbiome against bacterial overgrowth. Previous studies have revealed that HBV pre-S1 domain competing with NTCP blocks the uptake of the conjugated BAs (8) in HepG2-hNTCP cells and remarkably enhanced the expression of BA synthesis genes in HBV-infected human chimeric mice liver (9), suggesting that HBV binding to NTCP may increase the serum BA concentration. In contrary to this assumption however, we found that overall BA level did not change in NALT-CHB patients. In fact, transcriptomic data also did not support significant enhancement of hepatic CYP7A1 activity in CHB patients. Therefore, it is doubtful if the *de novo* synthesis of BAs is substantially enhanced in CHB patients. However, since HBV binding is known to deactivate FXRa (11), which suppress the expression of NTCP while maintains that of the BA exporter BESP (10, 11), therefore might explain the upregulation of NTCP and downregulation of BESP in CHB patients. This reciprocal expression change of BA importer and exporter suggested a compensatory accumulation of intrahepatic BAs in response to HBV infection.

Several studies have indicated that the composition of BAs is more relevant to progression of liver disease compared with the absolute level of total or individual BAs (33, 46–48). When looking at the detailed profile of BAs, we found that both CHB subgroups shared a decreasing ratio of unconjugated/conjugated BAs. As previous study have suggested, such change might be linked to the competitive binding of HBV to BA transporter NTCP (8, 9), which were mainly responsible for hepatic uptake of conjugated BAs, while OATP showed preference for unconjugated BAs (2, 49–51). Therefore, higher proportion of conjugated BAs was observed both in mouse with defective NTCP and human treated with NTCP inhibitor myrcludex B (2, 52). Our data, in agreement with those reports, suggested blocking of NTCP by HBV also caused relatively higher proportion of circulating conjugated BAs in CHB patients. To rule out the possibility of enhanced liver BA conjugation activity, we checked previous transcriptomic data (39) which revealed no significant changes of hepatic bile acid-CoA synthase (BACS) or bile acid-amino acid transferase (BAAT) in CHB patients (data not shown).

Other than hepatic factors, it is generally accepted that unconjugated BA levels can also be influenced by gut microbiome which harbors BSH enzymes for BAs deconjugation (23). Disrupted microbiome, for instance by means of antibiotic-treatments, resulted in host BAs profile dominated by conjugated species (53). Human microbiome studies also confirmed the impact of gut flora on BA compositions in various liver diseases, including liver cirrhosis, alcoholic liver disease, nonalcoholic fatty liver disease (NAFLD) (48, 54). Regarding this, we also found the gut microbiota diversity were lower and BSH expressing *Lactobacillus, Clostridium, Bifidobacterium, Enterobacteriaceae* were suppressed in CHB patients (Figure 4). This results is partially in agreement with previous microbiome study on CHB patients, which found lower abundance of *Bifidobacterium* and *Lactobacillus* (25). Therefore, caution should be taken to extrapolate findings by cellular or animal model in the scenario of HBV infection in human, in which both HBV-NTCP interaction and gut microbiome modulation were likely to influence BA compositions.

**Figure 5:**
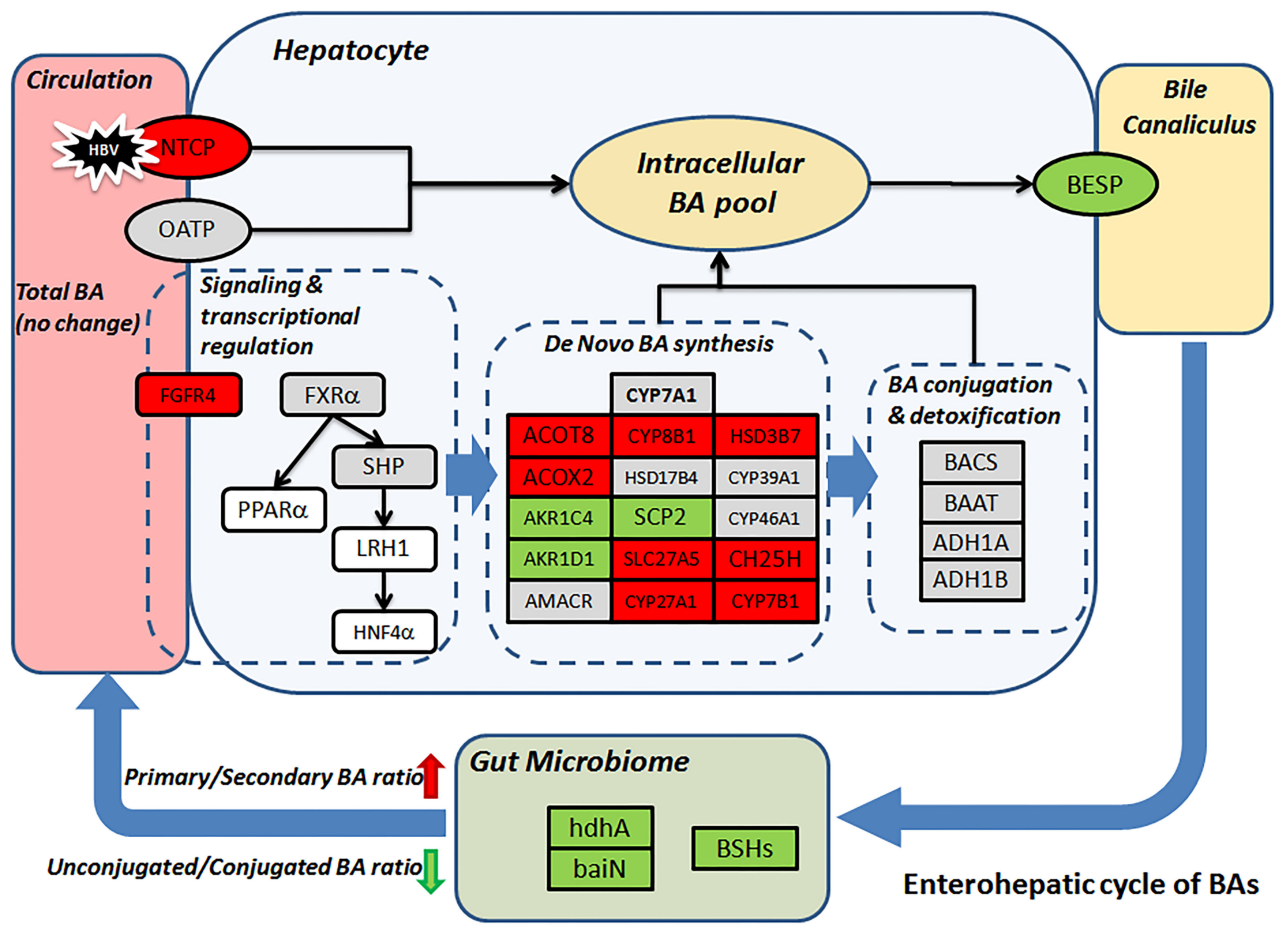
Proposed mechanism leads to the distinct BA profile in CHB patients. Change level of BA related genes (rectangular) according to transcriptomic data was visualized by color. Red/green represent up-/down-regulation, gray, and white indicate no-changes and missing data, respectively. BSHs, hdhA and baiN levels were predicted based on microbiome 16S sequencing data.

Gut microbiome modulation might also lead to the surged ratio of primary to secondary (p/s) BA ratio in the CHB-NALT patients. Previous microbiome profiling studies have found suppressed *Bifidobacterium, Clostridium*, and *Bacteroides* species in CHB patients (25, 55, 56). Similar observation was documented previously in cirrhotic patients which were also characterized by the deficiency of key gut microbiome taxa (48). Obviously, insufficient deconjugation activity will lead to lower level of substrates for secondary BA conversion, but we also found enzymes specifically responsible for secondary BA generation were impaired in CHB patients. These included 7-alpha-hydroxysteroid dehydrogenase (hdhA), which oxidizes the 7-alpha-hydroxy group of primary BA and is majorly found in *Bacteroides, Clostridia* and *Ruminococcus* spp (23, 57–60). One important member of hdhA producing bacteria, *B. fragilis* (58), was found significantly lower in CHB patients. The BA intermediates generated by HSDs are further processed by a group of bacterial enzymes encoded by the bile acid inducible (*bai*) operon, which catalyze only unconjugated bile acids and are highly conserved in both *Cl. scindens* and *Cl. hylemonae* strains (23, 61). In our analysis, we found 3-dehydro-bile acid delta-4,6-reductase (baiN) in the CHB patients was significantly lower than healthy controls. Taken together, the decreasing UCBA/CBA ratio and increasing p/s BA ratio indicate a deficient role of microbiome in the enterohepatic cycle of BAs in CHB patients, which is in line with previous data showing strikingly lower level of total fecal BAs in CHB patients even without cirrhosis (25). More importantly, such microbiota-mediated BA profile changes in NALT-CHB patients could be the prelude to more drastic changes in patients at advanced stages of CHB. During the completion of this study, another two studies of CHB patients with liver damages (ALT ≥ 40 IU/mL) and fibrosis have found interesting association of serum BA parameters with advanced fibrosis stages (62). Their data also confirmed the higher proportion of conjugated and primary BAs were in CHB patients and possible association with microbiome modulation (46, 62), which is in line with our founding. Moreover, previous study has observed that lower level of total, secondary, secondary/primary BA ratios, and higher primary BAs levels in feces correlated with the stage of liver cirrhosis, while serum primary BAs were also higher in patients with advanced cirrhosis (48). Major findings in our data and possible mechanism underlying the distinct profiles of CHB patients were summarized in Figure 5.

It is worthy to argue that there are likely a three-way mutual relationship among HBV, host BA metabolism and microbiota, as both BA and HBV also exerted their effects to remodel gut microbiota. As a group of amphipathic and bactericidal components, the BAs were well-documented as key regulator of gut microbial composition (23). Importantly, it is till recently the differential impact of different BA components on gut microbial species start to draw attention (63). Therefore, the distinct BA profiles could in turn contribute to the distinct microbial profiles in the CHB patients. For instance, the lower level of DCA in CHB patients might explain the higher percentage of *Bacteroides* which were shown to be inhibited by DCA produced majorly by *Firmicutes* (24). Previous studies have also reported gut flora alterations related to immune environment modulations during HBV infection. Mucosal immunity associated inflammatory cytokines (25, 64) such as the nuclear factor kappa B (NF-kB), tumor necrosis factor-alpha (TNF-a), and the secretory IgA (sIgA) were found significantly increased in CHB patients (65). Such alterations in host mucosal environment and immunity eventually affect intestinal microbial homeostasis. In short, our understanding of the complex relationship between HBV, host BA metabolism and microbiota is still incomplete thus warrant further investigation.

Currently, ALT and AST are two commonly used biomarkers to assess liver injury and to identify CHB patients for antiviral therapy. However, both markers lack organ specificity, and numerous studies have suggested that approximately 50–90% CHB patients with apparently normal ALT levels still have chronic inflammation by liver biopsy (66). Aminotransferases should therefore be argued as imperfect surrogate marker for active liver disease. Considering their higher liver specificity, BAs were proposed to predict liver necroinflammation (67). While BA signatures have been proposed as alternative biomarkers to gauge liver malfunctions to monitor end-stage liver diseases (30–33), we found the modulation of BA composition occurred at very early or mild stages of chronic HBV infection. Despite the highly heterogeneous profile of individual BA species found in all groups, our data showed that combinatory BA features such as total UCBAs, UCBAs/CBAs ratio and U/C ratio of CDCA could facilitate the diagnosis of chronic HBV infection with high accuracy. In parallel to our discovery, a recent study concluded that though limited changes in total serum BAs were found between NAFLD vs. non-NAFLD, or between nonalcoholic fatty liver vs. nonalcoholic steatohepatitis, the increased ratios of conjugated/unconjugated and primary/secondary BAs were associated significantly with liver fibrosis progression (47). Also, increased ratios of serum conjugated/unconjugated BAs were found in hepatocellular carcinoma patients (68). Therefore, we believe our preliminary data was encouraging, thus future longitudinal studies should be conducted in larger CHB cohorts with biopsy records to further determine whether BA signatures reflects fibrotic or necrotic development, or whether they prelude different clinical outcomes of CHB, including cirrhosis and hepatocellular carcinoma.

In summary, we found distinct patterns of serum BAs majorly featured by significant higher ratio of conjugated and primary BA species in CHB patients at early stages. These changes were likely to be the results of interaction of HBV with NTCP plus distinct gut microbiome alteration during HBV infection. Our findings provided a new insight into the complex relationship among HBV, BA and the gut microbiota in human, and suggested potential benefits of BA pathway or microbiota targeted interventions in the CHB patients.

## Data Availability Statement

The raw data supporting the conclusions of this article will be made available by the authors, without undue reservation.

## Ethics Statement

The studies involving human participants were reviewed and approved by Ethics Committee of the First Affiliated Hospital, School of Medicine, Zhejiang University. The patients/participants provided their written informed consent to participate in this study.

## Author Contributions

ZS, CH, MZ, and ZY: study concept and design. CH, YS, RW, JF, and YY: samples and clinical data collection. ZZ, KZ, and ZS: LC-MSMS experiments. ML and QN: microbiome profiling. ZS, CH, and ZY: analysis and interpretation of data. ZS, CH, and ZY: statistical analysis. ZC, MZ, and ZY: critical revision of the manuscript. ZS, CH, and ZY: drafting of the manuscript. All authors contributed to the article and approved the submitted version.

## Funding

This work was supported by the National Natural Science Foundation of China (82173578, 81871646, and 81400589), National Key Research and Development Program (2017YFC1200204 and 2017YFD0400300), The States S&T Projects of 13th 5 Year (2018ZX10302206), Zhejiang Provincial Medicine and Health Science Fund (2015KYAC031, 2016KYA083, and 2016147735), and Independent Project Fund of the State Key Laboratory for Diagnosis and Treatment of Infectious Disease.

## Conflict of Interest

The authors declare that the research was conducted in the absence of any commercial or financial relationships that could be construed as a potential conflict of interest.

## Publisher's Note

All claims expressed in this article are solely those of the authors and do not necessarily represent those of their affiliated organizations, or those of the publisher, the editors and the reviewers. Any product that may be evaluated in this article, or claim that may be made by its manufacturer, is not guaranteed or endorsed by the publisher.

## Abbreviations

(NTCP): Na+ taurocholate co-transporting peptide
(HBV): hepatitis B virus
(BA): bile acid
(CHB): chronic hepatitis B
(ALT): alanine aminotransferase
(AST): aspartate aminotransferase
(CHB-NALT): chronic hepatitis B with normal alanine aminotransferase
(CHB-AALT): chronic hepatitis B with abnormal alanine aminotransferase
(UPLC): ultra-performance liquid chromatography
(MS): mass spectrometry
(PICRUSt): phylogenetic investigation of communities by reconstruction of unobserved states
(BSH): bile salt hydrolases
(BSEP): bile salt export pump
(HBsAg): hepatitis B surface antigen
(HBeAg): hepatitis B e antigen
(GGT): gamma-glutamyl transpeptidase
(ALB): albumin
(UCBA): unconjugated bile acids
(CBA): conjugated bile acids
(GCBA): glyco-conjugated bile acids
(TCBA): tauro-conjugated bile acids
(PBA): primary bile acids
(SBA): secondary bile acids.
