# Supplementary material for: Distinct Bile Acid Profiles in Patients With Chronic Hepatitis B Virus Infection Reveal Metabolic Interplay Between Host, Virus and Gut Microbiome
Source: Front Med (Lausanne). 2021 Oct 4;8:708495. doi: 10.3389/fmed.2021.708495 (PMC8520922; doi:10.3389/fmed.2021.708495)
Supplement: Supplementary file 1 [file Data_Sheet_1.docx]

**Supplementary Data of manuscript:**

**Distinct bile acid profiles in patients with chronic hepatitis B virus infection reveal metabolic interplay between host, virus and gut microbiome**

**Table of Contents:**

## Supplementary Materials and Methods

- **Supplementary Tables**
- **Supplementary Figures**

**Supplementary Materials and Methods**

**UPLC-MRM−MS:**

All serum samples were separated by an ACQUITY UPLC BEH C18 2.1×100mm 1.7μm column (Waters) installed on an ultra-performance liquid chromatography (Class-I UPLC, Waters) couple with a triple-quadrupole mass spectrometry (Xevo-TQD, Waters) operated in the multiple reaction monitoring (MRM) scheme in the ESI-negative mode. The 21 min LC run at the flow rate of 0.4 mL/min was constructed by solvent A (0.01% FA) and solvent B (0.01% FA in acetonitrile) mixed according to gradient detailed in **Table S2**. The capillary voltage, source temperature, and desolvation temperature are −2 kV, 150 ºC, and 400 ºC, respectively. The desolvation, cone gas and collision gas (both were nitrogen) flow rates are 900, 50 L/h, and 0.25 mL/min, respectively. Instrument performance was constantly monitored by QC sample injections. Samples were kept at 4 ºC before injection, and column was operated under 50 ºC. The typical chromatogram can be found in **Figure S1**. Given the high stability of the UPLC and MS performance, successful alignment of LC-MS features of BAs between samples was achieved.

**Fecal bacterial genomic DNA extraction and 16S sequencing**

Fecal samples of 30 CHB-NALT patients and 30 healthy volunteers (**Table S4**) were used for microbiome profiling. Briefly, the first stool of the day was freshly collected in sterile containers, and immediately aliquoted and stored at -80 °C. a multiplexed amplicon library covering the 16S rDNA gene V4 region was generated from approx. 200 mg homogenized stool samples. Total bacterial DNA was isolated and purified using the QIAamp DNA Stool Mini Kit (QIAGEN, Germany). All 16S rRNA genes of distinct regions were amplified used specific primer (16S V4: 515F-806R) with the barcode. All PCR reactions were carried out with Phusion^®^ High-Fidelity PCR Master Mix (New England Biolabs). The library was sequenced on an Illumina HiSeq platform and 250bp paired-end reads were generated.

Sequences with ≥97% similarity were assigned to the same OTUs by Uparse software (v7.0.1001, http://drive5.com/uparse/). For each representative sequence, the SSUrRNA database was used to obtain the taxonomy information by the Mothur method and SILVA (<http://www.arb-silva.de/>). Alpha diversity was applied in analyzing complexity of species diversity for a sample through Shannon indices calculated with QIIME (v1.7.0). Nonparametric Wilcoxon rank-sum test was used to compare Beta diversity index between groups. LEfSe analysis identifies the most differentially abundant taxons at the classification level between groups using different LDA cutoff values (>2, >4). For all statistical tests for 16S rDNA data analysis, P values were adjusted for multiple hypothesis testing by Benjamini and Hochberg approach.

**Supplementary Tables:**

**Table S1**

**Definitions for clinical criteria**

| Alcoholic hepatitis | diagnosis of AH was based on Guidelines of prevention and treatment for alcoholic liver disease (2018). Briefly, a history of long-term alcohol consumption with laboratory finding of AST/ALT >2, plus elevated GGT and mean corpuscular volume. Ultrasound evidence including diffuse enhancement of near field echo in the hepatic region, which is stronger than that in the renal region; (ii) gradual attenuation of far field echo; (iii) unclear display of intrahepatic lacuna structure. |
| --- | --- |
| Non-alcoholic fatty liver disease | diagnosis of NAFLD was based on ultrasound evidence of a diffuse enhancement of near-field echo in the hepatic region and gradual attenuation of the far-field echo combined with any of the following: (1) unclear display of intrahepatic lacuna structure; (2) mild-to-moderate hepatomegaly with a round and blunt border; or (3) color Doppler ultrasonography showing a reduction in the blood flow signal in the liver or a blood flow signal that is difficult to display even when the distribution of the blood flow is normal |
| Liver cirrhosis | diagnosis of cirrhosis based on 1), previous diagnosis of cirrhosis; 2) sonographic evidence of liver nodularity combined with hepatic stigmata, spider angioma or splenomegaly; 3) portal hypertension; 4) enlarged spleen diameter, presence of esophageal varices or ascites; |
| Liver cancer | diagnosed based on pathological findings, clinical features or radiographic examinations according to American Association for the Study of the Liver Diseases (AASLD) guidelines, or previous diagnosis of liver cancer. |
| Gastrointestinal abnormalties | presentation of GI hematemesis, tarry stool and/or endoscopic signs of active bleeding or oozing from upper and/or lower GI varices; or the presence of variceal fibrin clots or red wale markings. |

**Table S2**

**LC-MRM analysis parameters for BA components**

| **BA** | **Parent M/Z** | **Daughter M/Z** | | **RT (min)** | **Dwell (ms)** | | **Cone (V)** | **Collision (V)** |
| --- | --- | --- | --- | --- | --- | --- | --- | --- |
| LCA | 375.27 | 375.30 | | 17.42 | 22 | | 90 | 10 |
| DCA | 391.27 | 391.31 | | 16.25 | 22 | | 88 | 10 |
| CA | 407.27 | 407.30 | | 13.59 | 22 | | 90 | 10 |
| GLCA | 432.33 | 73.90 | | 16.38 | 22 | | 76 | 44 |
| GDCA | 448.33 | 73.90 | | 14.80 | 22 | | 80 | 44 |
| GCA | 464.33 | 73.90 | | 10.71 | 22 | | 80 | 42 |
| TLCA | 482.33 | 79.95 | | 15.17 | 22 | | 110 | 70 |
| TDCA | 498.33 | 79.95 | | 12.01 | 22 | | 118 | 74 |
| TCA | 514.33 | 79.95 | | 8.59 | 22 | | 124 | 76 |
| UDCA | 391.27 | 391.31 | | 13.80 | 22 | | 88 | 10 |
| TUDCA | 498.33 | 79.95 | | 8.01 | 22 | | 118 | 74 |
| GUDCA | 448.33 | 73.90 | | 10.37 | 22 | | 80 | 44 |
| CDCA | 391.27 | 391.31 | | 16.09 | 22 | | 88 | 10 |
| TCDCA | 498.33 | 79.95 | | 11.35 | 22 | | 118 | 74 |
| GCDCA | 448.33 | 73.90 | | 14.13 | 22 | | 80 | 44 |
| **LC gradients** | | | | | | | | |
| **Steps** | **Time** | | **A%** | | | **B%** | | |
| 1 | 0 | | 80 | | | 20 | | |
| 2 | 2 | | 80 | | | 20 | | |
| 3 | 14 | | 60 | | | 40 | | |
| 4 | 18 | | 10 | | | 90 | | |
| 5 | 19 | | 10 | | | 90 | | |
| 6 | 19.1 | | 80 | | | 20 | | |
| 7 | 21 | | 80 | | | 20 | | |

**Table S3**

**Hepatic genes related to bile acid metabolism**

| **Function** | **Gene** | **Full name** |
| --- | --- | --- |
| BA biosynthesis (neutral pathway) | ACOT8 | acyl-CoA thioesterase 8 |
|  | ACOX2 | 3alpha,7alpha,12alpha-trihydroxy-5beta-cholestanoyl-CoA 24-hydroxylase |
|  | AKR1C4 (AK1C4) | 3alpha-hydroxysteroid 3-dehydrogenase / chlordecone reductase |
|  | AKR1D1 (AK1D1) | 3-oxo-5-beta-steroid 4-dehydrogenase |
|  | AMACR | alpha-methylacyl-CoA racemase |
|  | CYP7A1 | cholesterol 7alpha-monooxygenase |
|  | CYP8B1 | sterol 12-alpha-hydroxylase |
|  | HSD17B4 | (3R)-3-hydroxyacyl-CoA dehydrogenase |
|  | HSD3B7 | cholest-5-ene-3beta,7alpha-diol 3beta-dehydrogenase |
|  | SCP2 | sterol carrier protein 2 |
|  | SLC27A5 | solute carrier family 27 (fatty acid transporter), member 5 |
| BA biosynthesis (24/25-hydroxylase pathway) | CH25H | cholesterol 25-hydroxylase |
|  | CYP39A1 | 24-hydroxycholesterol 7alpha-hydroxylase |
|  | CYP46A1 | cholesterol 24-hydroxylase |
|  | CYP7B1 | 25/26-hydroxycholesterol 7alpha-hydroxylase |
| BA biosynthesis (acidic pathway) | CYP27A1 | cholestanetriol 26-monooxygenase |
| BA uptake | SLC10A1 | Sodium/bile acid cotransporter (Cell growth-inhibiting gene 29 protein) (Na(+)/bile acid cotransporter) (Na(+)/taurocholate transport protein) (Sodium/taurocholate cotransporting polypeptide) (Solute carrier family 10 member 1) |
|  | SLC10A2 | Ileal sodium/bile acid cotransporter (Apical sodium-dependent bile acid transporter) (ASBT) (Ileal Na(+)/bile acid cotransporter) (Ileal sodium-dependent bile acid transporter) (IBAT) (ISBT) (Na(+)-dependent ileal bile acid transporter) (Sodium/taurocholate cotransporting polypeptide, ileal) (Solute carrier family 10 member 2) |
|  | SLC10A4 | Sodium/bile acid cotransporter 4 (Na(+)/bile acid cotransporter 4) (Solute carrier family 10 member 4) |
|  | SLC10A7 | Sodium/bile acid cotransporter 7 (Na(+)/bile acid cotransporter 7) (Solute carrier family 10 member 7) |
|  | SLCO1A2 | Solute carrier organic anion transporter family member 1A2 |
| BA export | ABCB11 (BSEP) | Bile salt export pump |
| BA signaling | GPBAR1 (TGR5) | G-protein coupled bile acid receptor 1 (G-protein coupled receptor GPCR19) (hGPCR19) (Membrane-type receptor for bile acids) (M-BAR) (hBG37) (BG37) |
|  | NR1H4 (FXR) | Farnesoid X receptor |
|  | NR0B2 (SHP) | Nuclear receptor subfamily 0 group B member 2 (Small heterodimer partner) |
|  | FGFR4 (CD334) | Fibroblast growth factor receptor 4 |
| detoxification | ADH1A | Alcohol dehydrogenase 1A |
|  | ADH1B | Alcohol dehydrogenase 1B |
| BA conjugation | BAAT | bile acid-CoA:amino acid N-acyltransferase |
|  | SLC27A5 (BACS) | bile acid-CoA synthase |
|  | SULT2A1 | Bile salt sulfotransferase |

**Table S4**

**Baseline Characteristics of Study Subjects for stool examination**

|  | **HC (n=30)** | **CHB-NALT (n=30)** |
| --- | --- | --- |
| Age | 38.37r1.09 | 38.20±1.77 |
| HBV-DNA | \ | 6.88±3.63 E+06 |
| HBsAg | \ | Positive |
| HBeAg | \ | 64.65±30.37 |
| ALT | 26.10±3.30 | 36.55±7.12* |
| AST | 23.00±1.40 | 28.29±3.18* |

Note: All data were presented by mean±SEM. HC: healthy control group, CHB-NALT: ALT normal patient group. All subjects were males. HBsAg was detected by qualitative examination. The p values were determined by *t*-test, *p < 0.05.

**Table S5**

**Evaluation of using BA as biomarker for CHB**

| **Indicator** | **AUC** | **Std. Error** | | | **95% Confidence interval** | |
| --- | --- | --- | --- | --- | --- | --- |
| CA | 0.693 | 0.057 | | | 0.602 to 0.774 | |
| CDCA | 0.715 | 0.0568 | | | 0.625 to 0.794 | |
| DCA | 0.728 | 0.0605 | | | 0.639 to 0.805 | |
| LCA | 0.661 | 0.0564 | | | 0.569 to 0.745 | |
| UDCA | 0.757 | 0.0485 | | | 0.670 to 0.830 | |
| GCA | 0.623 | 0.0633 | | | 0.523 to 0.716 | |
| GCDCA | 0.547 | 0.0612 | | | 0.447 to 0.645 | |
| GDCA | 0.504 | 0.0683 | | | 0.405 to 0.603 | |
| GLCA | 0.534 | 0.0612 | | | 0.434 to 0.632 | |
| GUDCA | 0.526 | 0.0707 | | | 0.426 to 0.625 | |
| TCA | 0.619 | 0.0541 | | | 0.526 to 0.707 | |
| TCDCA | 0.641 | 0.0528 | | | 0.548 to 0.727 | |
| TDCA | 0.501 | 0.0576 | | | 0.408 to 0.594 | |
| TLCA | 0.574 | 0.0608 | | | 0.480 to 0.664 | |
| TUDCA | 0.817 | 0.0376 | | | 0.736 to 0.882 | |
| T_BA | 0.576 | 0.0566 | | | 0.483 to 0.666 | |
| T_UCBA | 0.775 | 0.0493 | | | 0.690 to 0.846 | |
| T_GBA | 0.542 | 0.057 | | | 0.449 to 0.634 | |
| T_TBA | 0.646 | 0.0527 | | | 0.553 to 0.731 | |
| T_CBA | 0.587 | 0.0565 | | | 0.494 to 0.676 | |
| R_U_C | 0.779 | 0.0505 | | | 0.694 to 0.850 | |
| R_G_T | 0.594 | 0.0567 | | | 0.500 to 0.682 | |
| R_uCA_cCA | 0.764 | 0.0486 | | | 0.678 to 0.837 | |
| R_uCDCA_cCDCA | 0.727 | 0.0559 | | | 0.639 to 0.805 | |
| R_uDCA_cDCA | 0.746 | 0.0509 | | | 0.658 to 0.821 | |
| R_uLCA_cLCA | 0.567 | 0.0572 | | | 0.473 to 0.657 | |
| R_uUDCA_cUDCA | 0.773 | 0.0479 | | | 0.688 to 0.845 | |
| T_pBA | 0.52 | 0.0583 | | | 0.427 to 0.612 | |
| T_sBA | 0.705 | 0.0539 | | | 0.615 to 0.785 | |
| R_p_s | 0.635 | 0.0583 | | | 0.542 to 0.721 | |
| R_CDCA_CA | 0.523 | 0.0576 | | | 0.430 to 0.615 | |
| R_CA_DCA | 0.612 | 0.065 | | | 0.519 to 0.700 | |
| R_CDCA_LCA | 0.506 | 0.0619 | | | 0.413 to 0.599 | |
| R_DCA_LCA | 0.589 | 0.0606 | | | 0.496 to 0.678 | |
| R_UDCA_DLCA | 0.511 | 0.071 | | | 0.418 to 0.603 | |
| R_UDCA_other | 0.588 | 0.0627 | | | 0.494 to 0.677 | |
| LCA% | 0.512 | 0.0617 | | | 0.420 to 0.605 | |
| CDCA_DCA% | 0.513 | 0.0589 | | | 0.420 to 0.605 | |
| LCA_CDCA_DCA% | 0.501 | 0.0646 | | | 0.408 to 0.593 | |
| CA_UDCA% | 0.501 | 0.0646 | | | 0.408 to 0.594 | |
| ALT | 0.75 | 0.0595 | | | 0.633 to 0.866 | |
| AST | 0.687 | 0.0594 | | | 0.570 to 0.803 | |
| ALP | 0.584 | 0.0642 | | | 0.458 to 0.710 | |
| GGT | 0.602 | 0.0675 | | | 0.470 to 0.734 | |
| TBil | 0.629 | 0.0611 | | | 0.509 to 0.748 | |
| **Logistic regression model** | | | | | | |
| Indicator | Coefficient | | Std. Error | | | P value |
| T_UCBA | -0.001 | | 0.000359 | | | 0.0055 |
| R_U_C | -2.16245 | | 0.68074 | | | 0.0015 |
| R_uCDCA_cCDCA | 1.70011 | | 0.65867 | | | 0.0098 |
| Constant | 3.1721 | |  | | |  |
| **ROC analysis of logistic regression model** | | | | | | |
| Cutoff criterion | | | | > 0.806 | | |
| Sensitivity | | | | 70.65 % | | |
| Specificity | | | | 85.71 % | | |
| Area under the ROC curve (AUC) | | | | 0.838 | | |
| AUC Std. Error | | | | 0.0401 | | |
| AUC 95% Confidence interval | | | | 0.759 - 0.917 | | |

**Supplementary Figures:**

**Figure S1,** representative base peak intensity chromatogram of BAs detected by LC-MSMS (1:TUDCA, 2:TCA, 3:GUDCA, 4:GCA, 5:TCDCA, 6:TDCA, 7:CA, 8:UDCA, 9:GCDCA,10:GDCA, 11:TLCA, 12:CDCA,13:DCA,14:GLCA, 15:LCA)


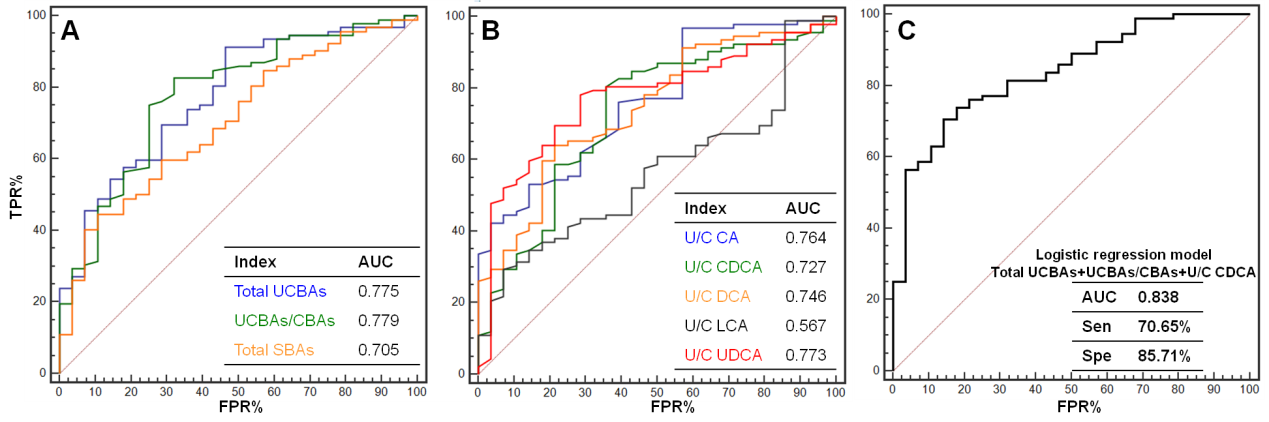


**Figure S2. Diagnostic performance of the BA markers for CHB monitoring.**

Comparison of unconjugated BA (UCBAs) level, UCBAs/CBAs ratios and total secondary BAs (SBAs) in prediction of CHB from health controls (**A**). Comparison of ROC curves of 5 major BA ratios (**B**). ROC curves of the final combinatory BA signatures to distinguish CHB from healthy controls (**C**). Sen: sensitivity; Spe: specificity.
